# Model-based estimation of measures of association for time-to-event outcomes

**DOI:** 10.1186/1471-2288-14-97

**Published:** 2014-08-09

**Authors:** Federico Ambrogi, Elia Biganzoli, Patrizia Boracchi

**Affiliations:** 1 Department of Clinical Sciences and Community Health, University of Milan, Via Venezian 1, 20133 Milan, Italy; 2 Fondazione IRCCS Istituto Nazionale Tumori, Via Venezian 1, 20133 Milan, Italy

**Keywords:** Survival analysis, Transformation models, Pseudo-values, Link functions, Numbers needed to treat

## Abstract

**Background:**

Hazard ratios are ubiquitously used in time to event applications to quantify adjusted covariate effects. Although hazard ratios are invaluable for hypothesis testing, other adjusted measures of association, both relative and absolute, should be provided to fully appreciate studies results. The corrected group prognosis method is generally used to estimate the absolute risk reduction and the number needed to be treated for categorical covariates.

**Methods:**

The goal of this paper is to present transformation models for time-to-event outcomes to obtain, directly from estimated coefficients, the measures of association widely used in biostatistics together with their confidence interval. Pseudo-values are used for a practical estimation of transformation models.

**Results:**

Using the regression model estimated through pseudo-values with suitable link functions, relative risks, risk differences and the number needed to treat, are obtained together with their confidence intervals. One example based on literature data and one original application to the study of prognostic factors in primary retroperitoneal soft tissue sarcomas are presented. A simulation study is used to show some properties of the different estimation methods.

**Conclusions:**

Clinically useful measures of treatment or exposure effect are widely available in epidemiology. When time to event outcomes are present, the analysis is performed generally resorting to predicted values from Cox regression model. It is now possible to resort to more general regression models, adopting suitable link functions and pseudo values for estimation, to obtain alternative measures of effect directly from regression coefficients together with their confidence interval. This may be especially useful when, in presence of time dependent covariate effects, it is not straightforward to specify the correct, if any, time dependent functional form. The method can easily be implemented with standard software.

## Background

Measures of disease frequency and measures of associations derived from them are among the basic building blocks of biostatistics and epidemiology. The appropriateness of the use of a specific measure of association may depend on the study objectives and design. Sometimes, however, the use of specific measures of association depends also on the statistical methods available for estimation. For example, in epidemiology, a debated subject concerns the use of odds ratios, estimated through logistic regression, in cohort studies of common outcomes [1,2].

When time-to-event outcomes are analyzed, the presence of censoring calls for specific methods of analysis [3]. The evaluation of the effect of a treatment in a controlled trial can be performed through the graphical display and comparison of Kaplan-Meier curves at selected times, when adjustment is not required. Otherwise, the measure of effect generally considered is the adjusted hazard ratio estimated by means of Cox proportional hazard model, ([4,5]).

However, the clinical literature in randomized controlled trials suggests the use of absolute measures of effect to assess the effects of a treatment, such as risk difference or the number needed to be treated, which are better suited than relative measures of effect for clinical decision support, see [6-10] among others. Schechtman highlights how relative measures are appropriate for summarizing the evidence while absolute measures for the concrete application in a clinical setting, [11].

The need for alternatives to hazard ratios, a relative measure of effect based on (instantaneous) incidence rates, is increasing in medical/epidemiological literature. In particular, the possibility to provide absolute measures of association computed using adjusted survival curves was explored in literature [12-14].

To precisely define the different measures of association in time-to-event applications, it is useful to distinguish between the risk of the event, *F*(*t*), i.e. the probability of a patient having the event over a defined follow-up time, and the event rate, *λ*(*t*), i.e. the number of events in a specified follow-up interval divided by the time at risk accumulated during the interval. The instantaneous hazard rate is obtained when the interval length approaches 0. The hazard rate at time *t* refers to the population survived until time t, while the risk refers to the whole population. The measures considered in this paper refer to ratios and differences between the risk of event of different groups of subjects. Let *F*_1_(*t*) and *F*_2_(*t*) be the event risk by *t* in two groups of subjects, (exposed and non exposed, standard treatment and new treatment), then we define the risk difference as *RD*(*t*)=*F*_1_(*t*)−*F*_2_(*t*). It is useful to translate *RD*(*t*) expressed as a percentage measure in a measure more sensible form a clinical perspective. To this end it is usual to use the number needed to be treated *NNT*(*t*)=1/*RD*(*t*) which is interpreted as the expected number of patients needed to be treated to avoid one additional death compared to the untreated. The measure has its roots in clinical trial literature and was extended in an epidemiological framework as the number needed to be exposed, *NNE*(*t*), i.e. the expected number of subjects to be exposed to have one additional event compared to the unexposed. In observational studies, an alternative definition of *NNE*(*t*) is the exposure effect among the unexposed, while the exposure impact number, *EIN*(*t*), describes the effect of removing the exposure among the exposed [15-17]. It is also interesting to define a relative risk, $\text{RR}(t)=\frac{F_{1}(t)}{F_{2}(t)}$, to be contrasted with the hazard ratio, $\text{HR}(t)=\frac{\lambda_{1}(t)}{\lambda_{2}(t)}$. The different measures of effect are in general time-varying. In certain situations, however, they are estimated as constant through follow-up, as it happens for example with the Cox proportional hazard model for the hazard ratio. When the measure is assumed to be constant during follow-up the time dependence is omitted (i.e., *HR*(*t*) is written as *HR*).

The purpose of this paper is to provide an outline of the methods generally adopted to estimate adjusted summary measures of associations, different from the hazard ratio, in time-to-event studies and to present a new method based on transformation models. The focus of this paper is not to provide guidance about which association measure should be used in different situations, but simply to provide an estimation method. Moreover, particular attention will be given to the estimate of adjusted *RD*(*t*). In fact, absolute measures of association are particularly advocated in survival analysis, to be combined with the generally used hazard ratio. A small simulation study is provided to show a preliminary evaluation of the properties of the different estimation procedures. Two examples are then developed. The first concerns literature data on a clinical trial on 506 prostate cancer patients [18]. The goal is to estimate the treatment *RD*(*t*) and *NNT*(*t*) with their confidence intervals. A comparison of the model based estimated *RD*(*t*) with that obtained with the classical corrected group prognosis method [14] is provided. The second application concerns an observational study of prognostic factors in primary retroperitoneal soft tissue sarcomas [19].

## Methods

### Computation of association measures

In many situations a researcher is interested in providing adjusted estimates of covariate associations with the outcome. In observational studies (involving no randomization) the exposure effect has to be adjusted for known confounders. Also in randomized controlled trials (RCT) the use of adjusted estimates is suggested for example to account for potential covariate imbalances or since prognostically relevant covariates were considered for a stratified randomization [20-22]. In these cases, Cox regression is widely used to adjust the estimated association between the covariate of interest and outcome for the other covariates.

For the purpose of illustration, let us consider a controlled trial, where the event of interest is death, investigating the efficacy of a new treatment (*T*=1) in comparison to a standard treatment (*T*=0). Two covariates such as age, A, and gender, G, are considered for adjustment. The multivariable proportional hazard Cox model can be specified as follows:

$$\lambda (t|T,A,G)=\lambda_{0}(t)\exp(\mathrm{\alpha T}+\mathrm{\beta A}+\mathrm{\gamma G})$$

where *t* is the time to event; *λ*(*t*|*T*,*A*,*G*) is the hazard function conditional to covariate values; *α*, *β* and *γ* are the regression coefficients; *λ*_0_(*t*) is the baseline hazard for a subject in the control group (*T*=0), 0 years old and female (*G*=0). The adjusted hazard ratio for the treatment, *HR* constant through follow-uptime, is simply obtained as exp(*α*). Using such a model, *RD*(*t*) or *NNT*(*t*) for the treatment can be obtained specifying a covariate pattern and the baseline risk. For example, the estimated *NNT*(*t*), conditional on being male 40 years old is:

(1) $$\frac{1}{Ŝ(t|T=1,A=40,G=1)-Ŝ(t|T=0,A=40,G=1)}.$$

where Ŝ(t|T=1,A=40,G=1) is the estimated survival probability for a male 40 years old in the experimental treatment group, given by ${\left[\;{Ŝ}_{0}(t)\right]}^{\text{exp}(\hat{\alpha }+\hat{\beta }\;40+\hat{\gamma })}$, while Ŝ(t|T=0,A=40,G=1) is the estimated survival probability for a male 40 years old, in the control group, given by ${\left[\;{Ŝ}_{0}(t)\right]}^{\text{exp}(\hat{\beta }\;40+\hat{\gamma })}$. ${Ŝ}_{0}(t)$ is the baseline survivor function from a Cox proportional hazards model estimated according to one of the available methods [23,24].

In order to obtain adjusted measures of association, different from the hazard ratio, the Cox proportional hazard model is used to estimate adjusted survival curves [25] as outlined in the following paragraphs.

### Average covariate method

The simplest approach for obtaining adjusted survival curves is the average covariate method. The mean values among the study patients of the covariates used for adjustment are plugged into the multivariable Cox proportional hazard model. Considering the above example, if the mean age of subjects in study is 45 and 30% males are included, the adjusted $\hat{\text{NNT}}(t)$ for the treatment will be $\frac{1}{Ŝ(t|T=1,A=45,G=0.3)-Ŝ(t|T=0,A=45,G=0.3)}$. The average covariate method was once popular and largely adopted, due to its simplicity, but it was severely criticized [25,26].

In fact it involves the averaging of categorical covariates, such as gender, which is difficult to understand. Moreover the method provides an estimate of the measure of effect for an hypothetical average individual and not a population averaged estimate.

### Corrected group prognosis method and developments

An alternative idea is the corrected group prognosis method (CGPM), [14,27,28]. In the following, the CGPM to estimate *RD*(*t*), as described by Austin [14], is outlined: 

• A Multivariable Cox (or fully parametric) regression is used for the treatment and the covariates.

• For each subject, the predicted survival probabilities, at the times of interest, are estimated using the multivariable model, assuming each subject is in the experimental treatment group; then the predictions are averaged;

• the same predictions are obtained and averaged assuming each subject is in the control group.

• the difference between the averaged predicted probabilities between experimental and control group is an estimate of the adjusted *RD*(*t*) for the experimental treatment at the specified times.

Pointwise confidence intervals of the obtained *RD*(*t*) estimates may be computed via bootstrap resampling [14]. For each bootstrap sample, i.e. a sample of the same size of the original one and randomly drawn with replacement from it, the *RD*(*t*) is computed according to the procedure outlined. A non parametric bootstrap 95% pointwise confidence interval is obtained resorting to the 2.5^*t* *h*^ and 97.5^*t* *h*^ percentiles of the obtained *RD*(*t*) bootstrap distribution.

**Figure 1:**
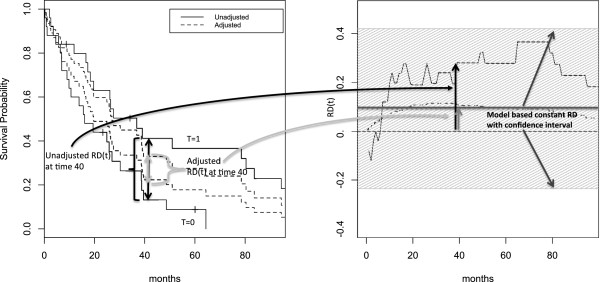
**Simulated data: *RD*(*t*) and Kaplan-Meier Curves.** Estimation of *RD*(*t*) associated with an hypothetical experimental treatment using artificial data simulated from a proportional hazard model (Details on the simulation are reported in the manuscript). The treatment effect is confounded by two covariates. The unadjusted Kaplan-Meier survival probabilities (- - - -) are reported together with the adjusted estimates (—–) obtained with the corrected group prognosis methods (left panel). The corresponding *RD*(*t*) estimates are reported in the right panel together with the model based constant *RD* estimate (with confidence interval).

A simulated example of the estimation of *RD*(*t*) in presence of confounding is exemplified in Figure 1.

The CGPM can be applied in principle to whatever regression model and an adequate model must be chosen. Considering, for example, the Cox regression model, in presence of time dependent covariate effects, an interaction of the covariates with a pre-specified function of time should be specified, in order to estimate *HR*(*t*) varying during follow-up time. It is important to remark that it is not always easy to specify an adequate model in presence of time dependent covariate effects. In fact it is not always obvious how to model the time dependence itself. In general simple functions of time (linear or logarithm) or more flexible alternatives are used, [29].

To allow the estimation the data set must be augmented as it is done for true time-dependent covariates [30]. It is to be remarked that, although the use of predicted values from regression models is simple from a practical point of view, the standard way to obtain summary measures of effect and their confidence interval is to use directly regression model coefficient estimates. The CGPM applied to the Cox model will be used for comparison with the method here proposed and described in the following section.

Laubender and Bender, [13], proposed different averaging techniques to estimate relevant impact numbers in observational studies using Cox model. For the purpose of illustration, let us consider the same example as before simply considering an exposure (*E*) instead of treatment. To obtain an estimate of *NNE*(*t*) it is possible to average predictions considering the subjects as if they were unexposed and as if they were exposed and taking the difference. As the distributions of the covariates used for adjusting are in general different in the exposed and unexposed groups, two different measures should be considered. Specifically, the estimate of the *NNE*(*t*) is obtained considering the unexposed subjects only, while *EIN*(*t*) is obtained considering the exposed subjects only. A comparison of the model based estimated *RD*(*t*) with that obtained through different averaging techniques, namely *NNE*(*t*) and *EIN*(*t*) [13,15], is provided in the second example. However, the focus of the paper is not the comparison of different averaging techniques which are provided only for illustrative purposes. In particular, only the estimates obtained through the averaging performed over the whole population are compared with those based on transformation models methods.

### Model-based estimates of association measures

Adjusted model-based estimates of measures of association can be obtained resorting to a general class of regression models used in Survival Analysis called transformation models [31].

#### Pseudo values

Considering the previous example, the transformation model can be written as *g*(*S*(*t*|*T*,*A*,*G*))=*g*(*S*_0_(*t*))+*αT*+*βA*+*γG*.

A possibility to estimate transformation models, using standard available software, is through pseudo-values [32]. The pseudo value is defined for each subject *i* at any time *t* and is given by

(2) $${\hat{\theta }}_{i}(t)=\mathrm{nŜ}(t)-(n-1){Ŝ}_{-i}(t)$$

where *n* is the sample size, Ŝ(t) is the survival probability based on the Kaplan-Meier estimator using the whole sample and ${Ŝ}_{-i}(t)$ is the survival probability obtained by deleting the *i* subject from the sample. When no censoring is present in the data, the pseudo values for subject *i* at time *t* is simply 1 if the subject is alive at *t*, while it is 0 if the event happened by *t*. Suppose to have an exposed male, 40 years old, which dies after 30 months of follow-up. The pseudo values computed at 12, 24 and 36 months are equal to 0, 0 and 1 respectively. The times at which the pseudo-values are computed are called pseudo-times.

When censoring is present in the data, pseudo-values are still defined for each subject (even those censored) and for each time, but the values may also be less than 0 or greater than 1 (See [33]; page 5310–11 for further details on the properties of pseudo-values).

In general, to allow inference on the entire survival curve, *M* (greater than 5) pseudo times are used, considering, for example, the quantiles of the unique failure time distribution. As *M* pseudo values are computed for each subject, an augmented data set is created with *M* observations for each subject.

#### Transformation models and association measures

The pseudo-values are then used as responses in a regression model for longitudinal data, where time is a covariate. As no explicit likelihood is available for pseudo-values, generalized estimating equations (GEE), [34], are used accounting for the correlation of the pseudo-values within each subject. The cluster robust variance-covariance is used for hypothesis testing using Wald tests. In general an independence working variance-covariance matrix can conveniently be used in the estimation process [32].

In order to model *g*(*S*_0_(*t*)), the transformed baseline survival function, the standard procedure is to insert in the regression model indicator functions for each pseudo-time. If all event times would be used to compute the pseudo-values, the insertion of indicator functions would result in a non parametric representation of the (transformed) baseline survival, as in the Cox model. In general only a small number of pseudo-times are used obtaining a parametric baseline representation. As an alternative, spline functions can be inserted in the regression model, as did [35] in a non-pseudo-values framework.

Considering for simplicity only two spline bases, the regression model of the example can be written as follows:

(3) $$g(\theta_{i}(t))=\phi_{0}+\phi_{1}B_{1}(t)+\phi_{2}B_{2}(t)+\alpha T_{i}+\beta A_{i}+\gamma G_{i}$$

where *B*_1_(*t*) and *B*_2_(*t*) represent the first and second spline bases for time t. For example, if a restricted cubic spline basis is used with three knots at *k*_1_,*k*_2_,*k*_3_, then *B*_1_(*t*)=*t* and $B_{2}(t)={\left(t-k_{1}\right)}_{+}^{3}-\frac{{\left(t-k_{2}\right)}_{+}^{3}(t_{3}-t_{1})}{\left(t_{3}-t_{2}\right)}+\frac{{\left(t-k_{3}\right)}_{+}^{3}(t_{2}-t_{1})}{\left(t_{3}-t_{2}\right)}$, where, for example, ${\left(t-k_{1}\right)}_{+}^{3}$ is equal to (*t*−*k*_1_)^3^ if *t*>*k*_1_, otherwise is 0. Knots are chosen at quantiles of the failure time distribution. In the case of 3 knots the quantiles commonly suggested are 0.1, 0.5 and 0.9, [36]. To choose the complexity of the spline the QIC, [37], an information criterion proposed for generalized estimating equations, can be used. A less formal strategy is the graphical comparison between the Kaplan-Meier marginal survival probability and the marginal probability obtained from the transformation model without covariates. Such a procedure will be used in the examples.

The first part of the model, *ϕ*_0_+*ϕ*_1_*B*_1_(*t*)+*ϕ*_2_*B*_2_(*t*), provides a parametric representation of the (transformed) baseline survival function, *g*(*S*_0_(*t*)), during follow-up time.

The coefficients *α*, *β* and *γ* represent the covariate effects expressed as differences in the Survival probability, transformed by *g* associated with a unit increase in the covariates. Let us consider such an issue in detail. When *g* is the logit link function, a proportional odds model is estimated. Accordingly, *α*, *β* and *γ* represent the logarithm of the ratio of the odds of surviving associated with the change of one unit in the covariates. Such an effect is constant through follow-up times. The exponentiation of the parameter estimates represent therefore the ratio of the odds of surviving. Similarly, the logarithmic link produces a proportional risks model and the *exp*(*α*), *exp*(*β*) and *exp*(*γ*) represent the ratio of the survival probabilities (Relative Risks, *RR*). The identity link produces a constant survival difference model: *α*, *β* and *γ* represent the adjusted differences in survival probabilities (risk differences, *RD*). A constant difference model through follow-up is often not practical as a model such that at the beginning of the follow-up the survival curves start at 1 and then, eventually, become different. However, it is to be noted that the first pseudo-time is never placed at time 0, but later on the follow-up time scale. In Figure 1 an example of the model based *RD* estimate with pointwise confidence intervals, constant through time, is reported in the right panel. The constant model estimated *RD* can be used to obtain a constant estimate of *NNT* by inversion. In the case of treatment T: $\hat{\text{NNT}}={\left[\hat{\alpha }\right]}^{-1}$. The value of 1 indicates the largest possible effect of *NNT*, while in correspondence of no covariate effect (*RD*=0) the *NNT* value is ±*∞*. The largest possible harmful effect is −1. Positive and negative values of *NNT* represent the expected number of patients needed to be treated for one additional patient to benefit and to be harmed, respectively.

In the case of the log-log link, *g*=*log*(−*log*(•)), *exp*(*α*), *exp*(*β*) and *exp*(*γ*) are the ratio between cumulative hazard functions associated with the change of one unit in the covariates. This ratio is equal to that of hazard functions, only in the proportional hazard case.

The method allows to estimate the measures of effect also for continuous covariates. For example, the evaluation of a biomarker effect measured on a continuous scale, without cutoffs, is still possible with this methodology.

The use of different link functions to obtain a particular measure of effect, is an established technique in binomial regression, where the use of non-canonical links, such as the logarithm, allows to obtain adjusted measures of impact different from the odds ratio, [1,38]. Wacholder, [39], is an excellent reference for deepen such aspects in the framework of logistic regression.

When there is evidence for time dependent effects of the covariates, the interaction between the covariates and the spline bases *B*_1_(*t*) and *B*_2_(*t*) are included in model (3).

() $$\begin{array}{lcrr} g(\theta_{i}(t)) & = & \phi_{0}+\phi_{1}B_{1}(t)+\phi_{2}B_{2}(t)+\alpha T_{i}+\beta A_{i}+\gamma G_{i} & \\ & & +\;\gamma_{1}B_{1}(t)T_{i}+\gamma_{2}B_{2}(t)T_{i}+\gamma_{3}B_{1}(t)A_{i} & \\ & & +\;\gamma_{4}B_{2}(t)A_{i}+\gamma_{5}B_{1}(t)G_{i}+\gamma_{6}B_{2}(t)G_{i} & \end{array}$$

In such a case, the estimated *g*-transformed survival probability differences change during follow-up time. In order to show the effect, varying in time, of a dichotomous covariate, for example treatment *T*, it is useful to adopt a graphical display, where the time is put on the horizontal axis while the function *exp*(*α*+*γ*_1_*B*_1_(*t*)+*γ*_2_*B*_2_(*t*)) is on the vertical axis (exponentiation is not used with the identity link; *RD*(*t*)=*α*+*γ*_1_*B*_1_(*t*)+*γ*_2_*B*_2_(*t*)). In this case the estimated *NNT*(*t*) is naturally varying through follow-up time and again obtain by inversion: *RD*(*t*)^−1^.

For a continuous covariate, such as Age, *A* in the example, it is possible to use a surface plot, where Age and time are on the *x* and *y* axis, while the *z* axis reports the covariate effect with respect to a reference value. It would also be possible to model Age effect with spline bases. In this case, the interaction between Age and time is obtained through tensor product spline bases of Age and time.

When a large number of pseudo-times is used, spline functions allow to model parsimoniously the baseline risk compared to indicator functions. This is particularly important for the modelling of time-dependent effects in connection to the different link functions. In principle when a covariate effect is constant using a specific link, it should be time-varying with the other links. No statistical evidence against a constant covariate effect for more than one link may only be due to lack of power. The problem can also be exacerbated by some multiple testing issue. Time-dependent effects selection depends therefore on the link transformation used. As a consequence, the adjusted effect of a covariate may be constant using a link function, but time-dependent using a different link.

Moreover, the fitted values of the different models selected for the different link functions are generally different, being equal only if the models are saturated. Traditionally, the strategy used in the application of transformation models such as (3) was to select the best fitting *g* transform, i.e., the transform where covariate effects are constant through time, see [40,41] as examples. The approach considered here is different. The interest is in using the *g*-transform which is the most informative for the clinical or biological counterpart. Generally the best fitting link function and the one selected by the researcher are not the same. Time dependent effects should therefore be expected in the model.

#### Pointwise confidence intervals

Approximate pointwise 95*%* confidence intervals are calculated from model results as in standard GLM/GEE modelling. The computation is easy when covariate effects are constant on the *g*-transformed scale. The cluster-robust variance-covariance matrix must be used. Using model (3) as example, the 95% CI for treatment, on the transformed *g* scale, will be

$$l_{\text{lower}},l_{\text{upper}}=\hat{\alpha }\pm 1.96\times \mathrm{st.error}(\hat{\alpha })$$

where $\mathrm{st.error}(\hat{\alpha })$ is the estimated cluster-robust standard error for the model parameter *α*. When *g* is the *log*, *logit* or *log*−*log* link function, the 95% CI for the treatment effect (respectively an RR, OR or HR) is [ *exp*.(*l*_*lower*_),*exp*(*l*_*upper*_)]. With the identity link, the 95*%* confidence intervals is [ *l*_*lower*_,*l*_*upper*_], without additional transformations, and the corresponding interval for *NNT* is [ 1/*l*_*upper*_,1/*l*_*lower*_].

A Clarification is necessary for the confidence interval of the NNT.

When the estimated constant *RD* is not statistically significant the confidence interval of *RD* includes 0. The limits of the confidence interval are one positive and the other negative. The resulting confidence interval for *NNT* should include infinity (*∞*), [42]:

$$95\mathrm{\%C.I.}=\left[-\infty ,1/l_{\text{lower}}\right]\cup \left[1/l_{\text{upper}},+\infty \right]$$

With time-varying covariate effects, the variance of the sum of a linear combination of different parameter estimates must be computed for the each of the follow-up times. For example, for treatment *T*, the variance of interest, at a specific time *t*, is that of the linear combination *α*+*γ*_1_*B*_1_(*t*)+*γ*_2_*B*_2_(*t*). Written in matrix terms the variance at time *t* is given by:

$$\begin{array}{c} \left(\begin{array}{ccc} 1 & B_{1}(t) & B_{2}(t) \end{array}\right)\;\left(\begin{array}{ccc} V(\alpha ) & \text{Cov}(\alpha ,\gamma_{1}) & \text{Cov}(\alpha ,\gamma_{2})\\ \text{Cov}(\alpha ,\gamma_{1}) & V(\gamma_{1}) & \text{Cov}(\gamma_{1},\gamma_{2})\\ \text{Cov}(\alpha ,\gamma_{2}) & \text{Cov}(\gamma_{1},\gamma_{2}) & V(\gamma_{2}) \end{array}\right)\;\left(\begin{array}{c} 1\\ B_{1}(t)\\ B_{2}(t) \end{array}\right) \end{array}$$

where *V*(•) stands for the cluster-robust variance, while *Cov*(•,•) stands for the cluster-robust covariance of two random variables. When the variances at the different times are calculated, the pointwise 95% CI can be computed as before.

#### Software implementation

The approach to censored data regression based on pseudo values was applied to regression models for the cumulative incidence functions in competing risks and for multi-state modeling [43], for the restricted mean [44] and for the survival function at a fixed point in time [45]. Implementation details and software can be found in Klein et al., [32], and Andersen and Perme, [46].

Software is available to compute pseudo values (macro %pseudosurv in SAS and function pseudosurv in R package pseudo[32]) Standard GEE tools available in SAS or R can be used for regression. In SAS the procgenmod allows to change link functions using the instructions FWDLINK and INVLINK. In R, the package geepack can be conveniently used, see [32] for details.

As an example of the R software implementation, the identity link is used: 

where the variable pseudo contains the pseudo values and the variable tpseudo the pseudo-times according to the software reported in [32]. The R function rcs of the package rms, [47], is used to compute restricted cubic spline bases. Each subject is represented by multiple rows in the data, one for each pseudo time. The records for each subject are identified by means of the variable id which is used to estimate the robust standard error by the geese function. Using the identity link function, the estimated coefficients can be interpreted as the adjusted *RD*(*t*) estimates.

## Results

The estimation of *RD*(*t*) through pseudo-values is evaluated through a simple simulation study. Moreover, two real examples are presented. The first example concerns a prostate cancer trial, well known in competing risks literature, to show a situation where proportional hazard fail to model the treatment effect during the whole follow-up. The second example regards the analysis of prognostic factors in an observational study of primary retroperitoneal soft tissue sarcoma patients. R software, [48], was used for the simulation and both the examples presented.

### Simulation

Data are generated to simulate Cox regression model, according to [13]. Suppose 100 persons are exposed (*Z*=1) and 100 unexposed (*Z*=0). A confounder *X* is generated normally distributed with mean 40 for *Z*=0 and 45 for *Z*=1 (standard deviations is 8 for both). Event times are generated according to an exponential proportional hazard model *λ*(*t*|*X*,*Z*)=*λe*^*l* *o* *g*(1.01)*X*+*l* *o* *g*(1.80)∗*Z*^. Censoring times are obtained in the same way adjusting the baseline hazard to have about 10*%* censoring. In such a situation the true exposure *RD*(*t*) may be calculated integrating out the covariate *X*:

(4) $$\begin{array}{c} \int_{-\infty }^{\infty }\;\;(S(t|Z=1,X)\;f(X|Z=1)-S(t|Z=0,X)f(X|Z=0))\text{dx} \end{array}$$

Pointwise confidence intervals for the CGPM are calculated using percentile bootstrap (200 bootstrap samples for each simulated data set). *RD*(*t*) is estimated by transformation models using pseudo values and the identity link. The baseline cumulative risk is modelled using a restricted cubic spline with 5 knots at 5*%*, 25*%*, 50*%*, 75*%* and 95*%* quantiles of the failure time distribution. In the pseudo value model the Z covariate is inserted either without and with an interaction with the baseline risk (i.e. estimating a constant *RD*, and a time-dependent *RD*(*t*) through follow-up time). The estimated *RD* and *RD*(*t*) at times 200, 400 and 600 are collected and compared with the true values. Such times are chosen to be sure that they are between the first and the last pseudo-times in all simulations. The results are analyzed in terms of bias, root mean squared error, average length and coverage of the 95*%* confidence intervals. The results are reported in Table 1.

**Table 1:** **Event times generated according to a Cox-exponential model with a confounder *X* and an exposure status *Z***

	**BIAS**		$\sqrt{\text{MSE}}$			
	PV	PV	Cox	PV	PV	Cox
	(*Z* td)	(*Z* const)	CGPM	(*Z* td)	(*Z* const)	CGPM
200	-0.0112	-0.0966	-0.0170	0.0756	0.1042	0.0580
400	-0.0047	-0.0681	-0.0145	0.0701	0.0785	0.0523
600	-0.0081	-0.0034	-0.0094	0.0528	0.0392	0.0376
	**Width**		**Coverage**			
	PV	PV	Cox	PV	PV	Cox
	(*Z* td)	(*Z* const)	CGPM	(*Z* td)	(*Z* const)	CGPM
200	0.2857	0.1527	0.2143	0.9430	0.3150	0.9480
400	0.2791	0.1527	0.1926	0.9560	0.5830	0.9340
600	0.1949	0.1527	0.1418	0.9350	0.9400	0.9320

CGPM was adopted using the Cox proportional hazard regression model. PV model was adopted with identity link. Pseudo times were fixed at quantiles 5%, 25%, 50%, 75%, 95% of the failure time distribution. The Z covariate was inserted in the PV model with an interaction with the baseline risk function modelled with a restricted cubic spline with 3 knots (*Z* td) giving an estimated risk difference varying through follow-up time, *RD(t)*, and without time-dependence to estimate a constant *RD* through follow-up time between the first and the last pseudo-times (*Z* const).

*RD*(*t*) estimated through the CGPM using the Cox proportional hazard regression model (the model used to generate the simulation data) is used as the benchmark estimation method.

The method based on pseudo-values with Z time dependent appears effective especially in terms of bias. Confidence interval coverage is good, although the width of the confidence intervals with pseudo-values is fairly large. It is interesting to observe the results of pseudo-values with the covariate *Z* not time dependent. In this case the estimated risk difference is constant through time, namely *RD*, a situation which can result from lack of power to detect the time-dependence of the *RD*(*t*). The simulation results appear very interesting for late follow-up times. At time 600, results are very similar to that of the Cox proportional model. However, the PV method with identity link and without time dependence estimating a constant *RD* leads to a strong undercoverage demonstrating that the estimation of a constant *RD* may be misleading.

The same simulation is performed generating times from a Cox model with a time-dependent effect for Z according to the following formula: *λ*(*t*|*X*,*Z*)=*λe*^*l* *o* *g*(1.01)*X*−*l* *o* *g*(0.95)∗*Z*∗log(*t*)^. In this second simulation the Cox model used with CGPM iss specified in two different ways. Specifically, the covariate *Z* is inserted with an interaction with log(*t*), the correct one, and with a restricted cubic spline of time with 3 knots. Namely the use of cubic splines for modelling time dependent effects was proposed by Hess, [29], allowing the study of possible covariate-time interactions without having to specify a specific functional form, using a limited number of parameters. The results are reported in Table 2.

**Table 2:** **Event times generated according to a Cox-exponential model with a confounder** ***X* and an exposure status** ***Z* with time-dependent effect**

	**BIAS**		$\sqrt{\text{MSE}}$			
	PV	Cox	Cox	PV	Cox	Cox
	*rcs*(*t*)	log(*t*)	*rcs*(*t*)	*rcs*(*t*)	log(*t*)	*rcs*(*t*)
200	-0.0290	0.0036	0.0151	0.0792	0.0689	0.1403
400	-0.0248	-0.0108	-0.0188	0.0730	0.0657	0.1119
600	-0.0185	-0.0134	-0.0242	0.0730	0.0657	0.1119
	**Width**		**Coverage**			
	PV	Cox	Cox	PV	Cox	Cox
	*rcs*(*t*)	log(*t*)	*rcs*(*t*)	*rcs*(*t*)	log(*t*)	*rcs*(*t*)
200	0.2899	0.2700	0.5162	0.9270	0.9530	0.9120
400	0.2747	0.2528	0.4211	0.9470	0.9500	0.9170
600	0.2158	0.1860	0.3256	0.9410	0.9410	0.9300

The effect of *Z* is multiplied by log(*t*). CGPM was adopted using the Cox proportional hazard regression model with the time dependent effect of *Z* specified in two ways: (1) with an interaction between Z and log(*t*), corresponding to the generating model; (2) with and interaction between *Z* and a restricted cubic spline of time (rcs(t)). PV model was adopted with identity link. Pseudo times were fixed at quantiles 5%, 25%, 50%, 75%, 95% of the failure time distribution. The Z covariate was inserted with an interaction with the baseline risk function modelled with a restricted cubic spline with 3 knots, giving an estimated risk difference varying through follow-up time, *RD*(*t*).

In this simulation the Cox model with the correct specification of the time dependent effect of *Z*, that is log(*t*), is used as benchmark estimation. When the time dependence of *Z* is modelled using the restricted cubic spline, the performance of the CGPM is less appealing, compared to the benchmark, regarding all the parameters considered into the simulation. The pseudo-value model is really a competitor in this situation. It is in particular interesting to observe the 95*%* confidence interval width. The transformation model using pseudo-values with identity link is a valuable alternative to the CGPM when the time dependent effect in the Cox model is unknown and modelled using a flexible method. Model checking is therefore very important and pseudo-values can be of help also in this case, see the work of Anderson and Perme, [46].

### Prostate cancer

Literature data on 502 prostate cancer patients, publicly available at the web site http://biostat.mc.vanderbilt.edu/wiki/Main/DataSets, (Byar & Greene prostate cancer data), treated with different doses of diethylstilbestrol in a randomized clinical trial, [18], were used to estimate the adjusted treatment effect (high versus low dose) on overall mortality. Seven covariates were used for adjustment, namely: age (0, < 75 years; 1, 75−80 years; 2, ≥ 80 years), weight index (0, ≥ 100;1, 80−99; 2, < 80), performance rating (0, normal; 1, limitation of activity), history of cardiovascular disease (0, no; 1, yes), serum haemoglobin (0, ≥ 12 g/100 ml; 1, 9-12 g/100 ml; 2, < 9 g/100 ml), size of primary lesion (0, < 30 *cm*^2^; 1, ≥ 30 *cm*^2^), and Gleason stage category (0, ≤ 10; 1, > 10). 483 patients with complete information on the seven covariates available were considered. 344 patients died: 149 for cancer; 139 for cardiovascular causes; 56 for other causes.

**Figure 2:**
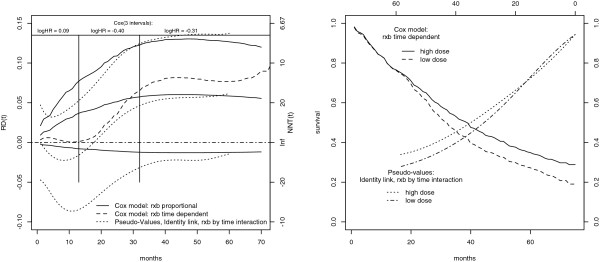
**Prostate Cancer Trial: Treatment *RD*(*t*).Left:** Estimate of treatment *RD*(*t*) and *NNT*(*t*) in the prostate cancer diethylstilbestrol trial by different methods. Continuous line: CGPM with the Cox proportional hazard model. The estimated *RD*(*t*) is increasing through follow-up time. Broken line: CGPM with a Cox model with a time dependent treatment effect (interaction treatment by time modeled with a B-spline with 1 knot at the median of the failure time distribution). There is no treatment effect until month 20, then *RD*(*t*) increases until month 40. Dotted line: estimate obtained using the transformation model with identity link. The *RD*(*t*) estimate is negative at the beginning of follow-up (harmful treatment) then becomes positive after about 20 months (beneficial treatment) reflecting the differential impact on cardiovascular and cancer deaths. On the top: three period Cox model used by Kay. The log hazard ratio is positive in the first period (0.09) [0−13], then becomes negative in the second (-0.40), (13−32], and in the third (-0.31), (32−*∞*), periods. The three period model was preferred to the overall Cox model according to likelihood ratio, accounting for non-proportional hazards. **Right:** estimated population averaged survival probability for the two intervention groups of the prostate cancer trial. The estimate from the time-dependent Cox model is reported from left to the right. The estimate from the transformation model with identity link is reported from right to the left. It is possible to observe a crossing of the survival curves.

The estimated *RD*(*t*) according to the CGPM using a Cox proportional hazard model [14] is reported in Figure 2. The estimated *RD*(*t*) is increasing through follow-up time, from 1*%* to 5*%*. Correspondingly the *NNT*(*t*) is about 100 at the beginning of the follow-up time and about 17 after 6 years follow-up. The 95*%* confidence interval is obtained using 2.5 and 97.5 percentiles of the bootstrap distribution of the estimated *RD*(*t*) (1000 bootstrap resamples). The *RD*(*t*) confidence interval includes 0.

The estimated *RD*(*t*) is based on a proportional hazard model. In fact there is no evidence for time-dependent treatment effect in Cox model according to Schoenfeld residuals. However, Kay, [18], carefully investigated the fit of the Cox model, dividing the time axis into three time interval: [0−13]; (13−32]; (32−*∞*). The log *HR* for treatment has a positive sign in the first period (0.09), then becomes negative (−0.40 and −0.31). Comparing by likelihood ratio the model fitted on three intervals with an overall survival model, evidence is found against the proportional *HR* assumption. In fact, cardiovascular deaths are more frequent than cancer deaths earlier during follow-up while, later on, cancer deaths are prevalent. Accordingly, the beneficial effect of treatment appears evident only after the first year of follow-up. The estimates obtained with the CGPM appear therefore distorted due to the use of a proportional hazard model.

As a model with jumps in covariate effects at specific times is not biologically plausible, a Cox regression model with an interaction between the treatment and a function of time is used (specifically a B-spline with 1 interior knot at the median of the failure time distribution). According to the work of Hess, [29], splines are used to model flexibly the possible treatment time dependent effect. The estimated *RD*(*t*) according to this flexible model is reported in Figure 2. The harmful initial treatment effect is not yet captured.

**Figure 3:**
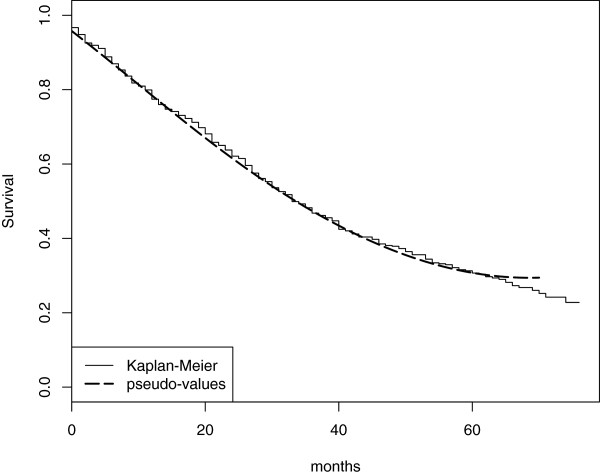
**Marginal Survival function.** Estimate of the marginal survival function for the prostate cancer data using a transformation model estimated with pseudo-values. 32 pseudo-times were considered. Each subject is therefore replicated 32 times in the dataset. The typical estimation of the baseline risk function is through indicator variables. In this case 32 coefficients should be included in the model. A B-Spline was instead used with one knot at the median of the unique failure time distribution resulting in 4 bases plus the intercept for modeling the baseline risk. The figure shows the estimated marginal survival with superimposed the Kaplan-Meier estimate.

A transformation model based on pseudo-values with identity link is used to estimate a possibly time-dependent treatment *RD*(*t*). In order to have about 10 events between each pseudo consecutive times, 32 pseudo-times are considered at quantiles of the unique failure time distribution. In this case B-splines are used to model the baseline cumulative risk. One knot, placed at the median of the failure time distribution, seems to be sufficient to model the marginal survival function, see Figure 3.

A backward selection procedure is used to select the time dependent effects for each covariate. The complete model has a total of 45 degrees of freedom, including the 8 covariates and their time dependent effects. The selected model exhibit a time dependent effect of history of cardiovascular disease, size of primary lesion and Gleason stage. There is no evidence for a time dependent treatment effect. The constant estimated *RD* is 2.2*%* with 95*%* confidence interval [ −3.5*%*;7.9*%*]. The corresponding constant estimate of *NNT* is about 45 patients to be treated for one patient to benefit with 95*%* confidence interval [ −*∞* to −28;13 to *∞*].

It is interesting, however, to compare the *RD*(*t*) estimated by the CGPM, which is by definition time dependent, with the one estimated through the transformation model letting the treatment effect varying in time. The results are reported in Figure 2 and summarized in Table 3. The treatment effect is harmful during the first year, while the benefits appear afterwards in agreement with the analysis of Kay [18] further refined using competing risks.

**Table 3:** ***R*** ***D*** **(*t*) estimated with different methods at months 13, 32 and 60**

	**RD**		
	**13**	**32**	**60**
*RD*(*t*): Cox prop	3.7*%*; (−1.2*%*;7.7*%*)	5.7*%*; (−1.2*%*;12.3*%*)	5.8*%*; (−1.2*%*;12.7*%*)
*RD*(*t*): Cox TD	0.1*%*; (−6.8*%*;6.5*%*)	6.5*%*; (−1.5*%*;14.1*%*)	7.7*%*; (−0.5*%*;15.6*%*)
*RD*: PV identity Z const	2.2*%*; (3.5*%*;−7.9*%*)	2.2*%*; (3.5*%*;−7.9*%*)	2.2*%*; (3.5*%*;−7.9*%*)
*RD*(*t*): PV identity Z TD	−1.6*%*; (−8.5*%*;5.3*%*)	4.6*%*; (−3.2*%*;12.4*%*)	6.2*%*; (−1.4*%*;13.7*%*)

Cox prop: CGPM with a Cox model without treatment time dependent effect; Cox TD: CGPM with a Cox model with treatment time dependent effect; PV identity Z const: Pseudo-value model with identity link and constant treatment effect *RD*; PV identity Z TD: Pseudo-value model with identity link and time dependent treatment effect. B-splines were used to model treatment time dependent effects

In the right panel of Figure 2 are also reported the averaged survival probabilities obtained by the CGPM using the Cox model and the transformation model both with the time-dependent effect of treatment. The plot of the averaged survival probabilities is an important completion of the *RD*(*t*) plot. In fact *RD*(*t*), as usual for the effect measures, is reducing two numbers to a single number.

The prostate cancer data outline a scenario in which the proportional hazard assumption for the treatment effect is not tenable during all follow-up times. Data on prostate cancer should be actually analyzed accounting for competing risks. When a non competing risks survival analysis is performed, CGPM applied to the Cox model without a time dependent treatment effect gives an estimate of the *RD*(*t*) increasing during follow-up until reaching a plateau. At the same time, the constant estimate *RD* obtained using PV provides a distorted estimate constant through follow-up. CGPM applied to the Cox model with a time dependent treatment effect provides an *RD*(*t*) estimate not yet capturing the initial harmful treatment effect. The time-dependent estimate obtained from the pseudo-value model is instead effective in describing the treatment effect during follow-up time: harmful at the beginning, when cardiovascular deaths are more frequent, beneficial later on when cancer deaths are more frequent

### Primary retroperitoneal soft tissue sarcoma

**Figure 4:**
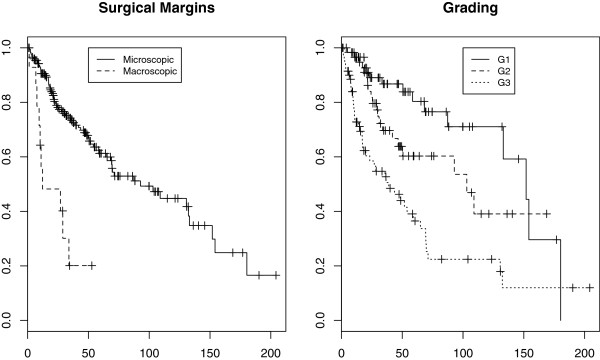
**Kaplan-Meier overall survival curves.** Patients with retroperitoneal soft tissue sarcoma. Left panel: Surgical Margins. All patients underwent surgery but some of them (14), especially those with advanced disease status, failed to have complete resection. Right panel: Grading (1: 60; 2: 62; 3: 70).

Retroperitoneal soft tissue sarcoma (STS) is an uncommon disease. Histologic grade and completeness of macroscopical resection are considered to be the major prognosticators for survival, while histologic type, size and age are more debated. One hundred and ninety two patients with retroperitoneal STS admitted to National Cancer Institute in Milan, consecutively, between 1985 and 2007 are considered. Patients are without evidence of locoregional recurrence or distant metastasis. The median follow-up is 54.8 moths (IQR: 25-104). Among the 192 patients included in the case series, 78 died during follow-up. For a complete description of patients characteristics see [19]. Kaplan-Meier Survival estimates for resection margin and grading are reported in Figure 4. The covariate resection margins is strongly unbalanced in this case series. No evidence for time dependent covariate effects is found using Schoenfeld residuals. A restricted cubic spline with 3 knots, for a total of 2 bases, is used to model the marginal transformed baseline cumulative survival. The fit and the monotonicity of the estimated baseline cumulative risk is controlled by visual inspection [data not shown]. A backward procedure is used for time-dependent covariate selection, fixing the significance at a conservative 0.01 level. Using the *cloglog* link function, no evidence for time dependent effects is found. Considering the *log* link, the effect of Grading appears time-varying, while using the *identity* link, Grading and resection margins are modelled varying in time. For the sake of completeness, the estimates of the association between resection margins and survival using the pseudo value model and a *cloglog* link are compared with the results from the Cox model. The cumulative constant *HR* is 2.4 (95% *CI*: 1.38−4.31) with pseudo-values. Such an association appears conservative compared to that estimated through Cox regression: *HR*=4.19 (95% *CI*: 2.04−8.59). In general it is possible to note an attenuation of estimated effects using PV and the *cloglog* scale, compared to the Cox model: in the case of resection margins the attenuation is quite important. Clearly, the pseudo-value model with *cloglog* link is of no interest in this setting, while it has an interest for model checking in Cox regression, [46].

**Figure 5:**
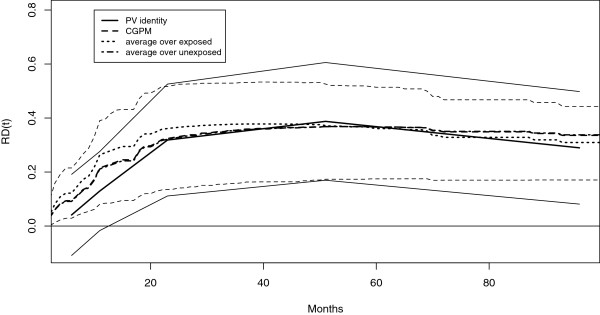
**Retroperitoneal soft tissue sarcoma: Margins.***RD*(*t*) of extension of resection margins estimated using different methods. PV identity: estimated effect using pseudo values, identity link and interaction between time and margins. Five pseudo times were used at times 6, 11, 23, 51, 96, corresponding to quantiles 10*%*, 25*%*, 50*%*, 75*%*, 90*%* of the failure time distribution. CGPM: Corrected group prognosis method using a proportional hazard Cox regression model. The averaging is performed on the whole sample of patients. Other two measures are reported: (1) average over exposed: obtained averaging only on patients with macroscopic resection margins (*EIN*(*t*)); (2) average over unexposed: obtained averaging only on patients without microscopic resection margins (*NNE*(*t*)).

Also the constant *RR* estimated using pseudo values and *log* link appears conservative: *RR* = 1.3 (95% *CI*: 1.03−1.71). A comparison of the estimated *RD*(*t*) obtained with pseudo values and *identity* link with the estimates obtained using the CGPM is reported in Figure 5. Different averaging procedures are also reported. The estimated *RD*(*t*) and point-wise confidence intervals using the different methods are in close agreement. The different averaging procedures yield quite similar results in this case. According to pseudo-values, the *RD*(*t*) reaches a maximum value at 60 months and is about 40*%*.

**Figure 6:**
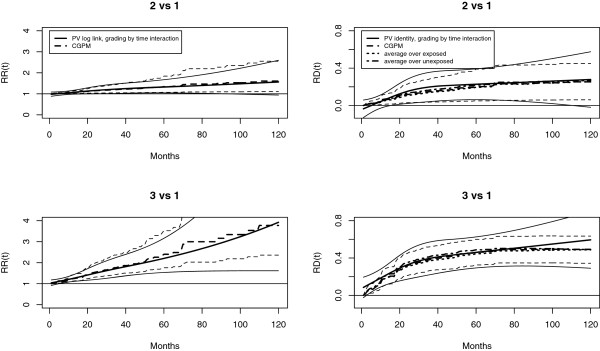
**Retroperitoneal soft tissue sarcoma: Grading.** The effect of the different Grading categories on the *RR*(*t*) and *RD*(*t*) scales are reported, using different estimation methods. CGPM applied to a proportional hazard Cox model; PV with *log* and *identiy* links and interaction between time and grading. The estimates obtained with the different techniques appears quite in agreement. No evidence for time dependent effects in the Cox model was found.

According to Cox regression the *HR* of grading 2 vs 1 is 2.56 (1.27−5.16). The estimate obtained through pseudo-values and *cloglog* link is 2.34 (1.27−4.32). There is still an attenuation but not as huge as in the case of margins. However, considering grading 3 vs 1 the attenuation effect is more evident: the *HR* from Cox regression is 6.49 (3.31-12.75), while using pseudo-values is 4.88 (2.54-9.39). The *RR*(*t*) and *RD*(*t*) are reported in Figure 6. After 8 years the *RR*(*t*) is 1.6 and 3.1, and the *RD*(*t*) is 36*%* and 59*%*, for 2 vs 1 and 3 vs 1 contrasts, respectively. Also in the case of grading, the estimates obtained using pseudo-values and those obtained using different averaging procedures from the Cox regression model appear in good agreement. The quantification of effects through risk differences may be very important for the clinical management of the disease.

## Discussion

In survival analysis the adjusted measure of association everywhere adopted is the hazard ratio. Although the efficiency of the hazard ratio makes it attractive for hypothesis testing, it may not carry the most useful information for clinicians/biostatisticians. Schechtman, [11], suggests using also absolute measures in conjunction with relative measures of covariate-outcome association. To provide adjusted measures of association different from the hazard ratio, a simple strategy is going through the calculation of the predicted probabilities of event for an “average” subject, the so called “average covariate method”. Such a procedure estimates the measures of effect for an hypothetical average subject and not population averaged estimates. An alternative idea to provide adjusted summary measures of effect is the corrected group prognosis method [27,28]. Extending this idea, using the concept of counterfactuals, Laubender and Bender [13] proposed methods for computing such population measures taking into account the confounder distribution. Bootstrap is then used to obtain pointwise confidence intervals. Such approaches are particularly appealing as they may adopt the Cox regression model which is widely accepted in medical literature.

However the Cox model may easily not be the best regression procedure to be applied, simply because of the assumption of proportional hazards. In fact, in presence of time dependent effects Cox regression may be less appealing. This is demonstrated here through a simple simulation. When time-dependent effects in the Cox model are specified using well-known flexible methods, [29], without committing to a specific functional form, the estimates are not optimal, especially in terms of efficiency. In these circumstances the results of the simple simulation presented here, suggest that the use of the pseudo-value model may represent a valid alternative to the CGPM. The simulation is not exhaustive and more work is needed to fully understand the properties and the relationships among the different estimation methods.

Considering a similar problem in the context of logistic regression, Gehrmann and colleagues, [49], concluded that the CGPM applied to logistic regression is the preferred method to estimate *RD* and *NNT* adjusted for covariates compared to binomial, Poisson and linear regression methods that directly estimates the *RD* (similar to pseudo-values with identity link) even if the fitted response function differs from the true response function. The context of time-to-event outcomes is more complex than that of logistic regression especially for the problem of time dependent effects. Whether similar results hold for the Cox model has therefore to be further explored thorough a series of simulation studies. In any case, when using the CGPM, the basic model used to obtain event probabilities during follow-up has to be adequate. This means, for example, that the proportional hazard Cox regression should not be applied if the proportional hazard assumption is not satisfied.

In clinical literature, results of statistical analysis are commonly reported in terms of regression coefficients and their confidence intervals. Applied survival analysis resorts entirely to the Cox model which is a particular case of transformation models. Transformation models include also the accelerated failure time models providing a variety of measures of effects to be considered.

It is to be noted that, additive and multiplicative-additive hazard regression models, [31] are not comprised in this class. The estimated coefficients are differences of hazard rates rather than ratios. These models were mainly proposed to improve the fitting where the proportional hazards model is not adequate or to check for proportional hazard assumptions. Moreover, the measures of impact provided are still based on hazards.

In this work a simple approach to obtain point and interval estimates of association measures, by using transformation models through suitable link functions, is presented. The general technique of estimation based on pseudo-values proposed by Andersen and colleagues [43] is used as it is simply implementable with standard software.

Other techniques could have been considered to estimate the transformation models. In this context, it is of particular interest the estimate of the baseline survival function to model time dependent effects. Therefore, semi-parametric techniques (see for example [50]) are not of interest here. Maximum likelihood (ML) estimation can however be conveniently used. Royston and Parmar proposed ML for transformation models with *cloglog* and *logit* links, [51], which are, however, the links of less interest here. Moreover as the pseudo-value is defined between the first and the last pseudo times (which are not the first and last event times) it still makes sense to have a constant *RD* model. In fact considering the whole follow-up time interval, and especially the beginning of the follow-up, *RD*(*t*) estimates should instead always be time-dependent.

From the methodological viewpoint, the only difference introduced in the presented examples with respect to standard applications of the pseudo-value model is the use of spline functions to estimate the transformed baseline survival. This modification is without practical efforts, considered the wide availability of software to compute spline bases. From the modeling point of view, care must be paid to the monotonicity of the estimated transformed baseline survival function. In general, provided the number of knots is limited, no problems of non monotonicity were observed.

Another issue concerns the possible simultaneous use of different association measures estimated through different link functions in a transformation model. In such a case, practitioners must be aware that, due to lack of power, not all time dependent effects can be correctly specified, and likely the different models cannot hold simultaneously. In such a case, if different measures are of interest, different models can be used simultaneously only if the results are in agreement with each other.

From a theoretical point of view, *cloglog* and *logit* links guarantee that the estimated probabilities are within the range 0-1 but this is not guaranteed if *log* or *identity* links are used with standard software. Research is in progress to face this issue.

## Conclusions

The use of different link functions in transformation models has been studied by several authors simply investigating the goodness of fit of the different links [40,41].

The alternative perspective considered here, evaluates the use of different links with the goal of providing suitable measures of association between covariates and outcome. As a consequence, when a specific link function is chosen, it should be expected the need to include time dependent covariate effects into the model.

Transformation models estimated through pseudo-values appear an easily implemented alternative to the available approaches mainly based on Cox proportional hazard model to obtain adjusted measures of association eventually time-dependent also for continuous covariates.

## Competing interests

The authors declare that they have no competing interests.

## Authors’ contributions

FA drafted the manuscript, designed the study and performed the analyses. EB participated in the design of the study and revised critically the manuscript. PB participated in the design of the study and revised critically the manuscript. All authors read and approved the final manuscript.

## Pre-publication history

The pre-publication history for this paper can be accessed here:

http://www.biomedcentral.com/1471-2288/14/97/prepub

## References

[B1] Greenland S **Model-based estimation of relative risks and other epidemiologic measures in studies of common outcomes and in case-control studies** Am J Epidemiol 2004 160 4 301 305 15286014 10.1093/aje/kwh221

[B2] McNutt LA Wu C Xue X Hafner JP **Estimating the relative risk in cohort studies and clinical trials of common outcomes** Am J Epidemiol 2003 157 10 940 943 12746247 10.1093/aje/kwg074

[B3] Marubini E Valsecchi MG Analysing Survival Data from Clinical Trials and Observational Studies. Statistics in Practice 2004 Chichester: Wiley

[B4] Cox D **Regression models and life-tables (with discussion)** J R Stat Soc B 1972 34 557 565

[B5] Cox D **Partial likelihood** Biometrika 1975 62 269 276

[B6] Laupacis A Sackett DL Roberts RS **An assessment of clinically useful measures of the consequences of treatment** N Engl J Med 1988 318 26 1728 1733 3374545 10.1056/NEJM198806303182605

[B7] Boracchi P Mezzanotte G Mariani L Valagussa P Marubini E **Clinically useful measures to assess the effectiveness of treatments: hints for a proper choice with special emphasis on cancer research** Tumori 1990 76 1 1 9 2321266 10.1177/030089169007600101

[B8] Jaeschke R Guyatt G Shannon H Walter S Cook D Heddle N **Basic statistics for clinicians: 3. Assessing the effects of treatment: measures of association** CMAJ 1995 152 3 351 357 7828099 PMC1337533

[B9] DiCenso A **Clinically useful measures of the effects of treatment** Evid Based Nurs 2001 4 2 36 39 11708053 10.1136/ebn.4.2.36

[B10] Case LD Kimmick G Paskett ED Lohman K Tucker R **Interpreting measures of treatment effect in cancer clinical trials** Oncologist 2002 7 3 181 187 12065789 10.1634/theoncologist.7-3-181

[B11] Schechtman E **Odds ratio, relative risk, absolute risk reduction, and the number needed to TreatWhich of these should we use?** Value Health 2002 5 5 431 436 12201860 10.1046/J.1524-4733.2002.55150.x

[B12] Austin PC Laupacis A **A tutorial on methods to estimating clinically and policy-meaningful measures of treatment effects in prospective observational studies: a review** Int J Biostat 2011 7 1 1 32 10.2202/1557-4679.1285 PMC3404554 22848188

[B13] Laubender RP Bender R **Estimating adjusted risk difference (RD, and number needed to treat (NNT, measures in the Cox regression model** Stat Med 2010 29 7–8 851 859 20213710 10.1002/sim.3793

[B14] Austin PC **Absolute risk reductions and numbers needed to treat can be obtained from adjusted survival models for time-to-event outcomes** J Clin Epidemiol 2010 63 1 46 55 19595575 10.1016/j.jclinepi.2009.03.012

[B15] Bender R Kuss O **Methods to calculate relative risks, risk differences, and numbers needed to treat from logistic regression** J Clin Epidemiol 2010 63 1 7 8 19762212 10.1016/j.jclinepi.2009.07.007

[B16] Bender R Kuss O Hildebrandt M Gehrmann U **Estimating adjusted nnt measures in logistic regression analysis** Stat Med 2007 26 30 5586 5595 17879268 10.1002/sim.3061

[B17] Austin PC **Different measures of treatment effect for different research questions** J Clin Epidemiol 2010 63 1 9 10 19837563 10.1016/j.jclinepi.2009.07.006

[B18] Kay R **Treatment effects in competing-risks analysis of prostate cancer data** Biometrics 1986 42 1 203 211 3521753

[B19] Ardoino I Miceli R Berselli M Mariani L Biganzoli E Fiore M Collini P Stacchiotti S Casali PG Gronchi A **Histology-specific nomogram for primary retroperitoneal soft tissue sarcoma** Cancer 2010 116 10 2429 2436 20209615 10.1002/cncr.25057

[B20] Hauck WW Anderson S Marcus SM **Should we adjust for covariates in nonlinear regression analyses of randomized trials?** Control Clin Trials 1998 19 249 256 9620808 10.1016/s0197-2456(97)00147-5

[B21] Senn SJ **Covariate imbalance and random allocation in clinical trials** Stat Med 1989 8 467 475 2727470 10.1002/sim.4780080410

[B22] International Conference on Harmonization **Harmonised tripartite guideline: statistical principles for clinical trials** Stat Med 1999 18 15 1905 1942 Cited By (since 1996) 214 10532877

[B23] Fleming TH Harrington DP **Nonparametric estimation of the survival distribution in censored data** Comm Stat 1984 13 2469 2486

[B24] Kalbfleisch JD Prentice RL The Statistical Analysis of Failure Time Data (Wiley Series in Probability and Statistics) 2002 New York: Wiley-Interscience

[B25] Ghali WA Quan H Brant R van Melle G Norris CM Faris PD Galbraith PD Knudtson ML **Comparison of 2 methods for calculating adjusted survival curves from proportional hazards models** JAMA 2001 286 12 1494 1497 11572743 10.1001/jama.286.12.1494

[B26] Nieto FJ Coresh J **Adjusting survival curves for confounders: a review and a new method** Am J Epidemiol 1996 143 10 1059 1068 8629613 10.1093/oxfordjournals.aje.a008670

[B27] Chang IM Gelman R Pagano M **Corrected group prognostic curves and summary statistics** J Chronic Dis 1982 35 8 669 674 7096530 10.1016/0021-9681(82)90019-4

[B28] Makuch RW **Adjusted survival curve estimation using covariates** J Chronic Dis 1982 35 6 437 443 7042727 10.1016/0021-9681(82)90058-3

[B29] Hess KR **Assessing time-by-covariate interactions in proportional hazards regression models using cubic spline functions** Stat Med 1994 13 10 1045 1062 8073200 10.1002/sim.4780131007

[B30] Therneau TM Grambsch PM Modeling Survival Data: Extending the Cox Model 2010 New York: Springer

[B31] Martinussen T Scheike TH Dynamic Regression Models for Survival Data (Statistics for Biology and Health) 2006 New York: Springer

[B32] Klein JP Gerster M Andersen PK Tarima S Perme MP **SAS and R functions to compute pseudo-values for censored data regression** Comput Methods Programs Biomed 2008 89 3 289 300 18199521 10.1016/j.cmpb.2007.11.017 PMC2533132

[B33] Perme MP Andersen PK **Checking hazard regression models using pseudo-observations** Stat Med 2008 27 25 5309 5328 18712781 10.1002/sim.3401 PMC2749183

[B34] Liang KY Zeger SL **Longitudinal data analysis using linear models** Biometrika 1986 73 13 22

[B35] Royston P Parmar MK **Flexible parametric proportional-hazards and proportional-odds models for censored survival data, with application to prognostic modelling and estimation of treatment effects** Stat Med 2002 21 15 2175 2197 12210632 10.1002/sim.1203

[B36] Harrell FE **Regression Modeling Strategies** 2001

[B37] Pan W **Akaike’s information criterion in generalized estimating equations** Biometrics 2001 57 120 125 11252586 10.1111/j.0006-341x.2001.00120.x

[B38] Hardin JW Hilbe J Generalized Linear Models and Extensions 2001 College Station, Texas: Stata Press

[B39] Wacholder S **Binomial regression in GLIM: estimating risk ratios and risk differences** Am J Epidemiol 1986 123 1 174 184 3509965 10.1093/oxfordjournals.aje.a114212

[B40] Bennett S **Analysis of survival data by the proportional odds model** Stat Med 1983 2 2 273 277 6648142 10.1002/sim.4780020223

[B41] Wei LJ **Testing goodness of fit for proportional hazards model with censored observations** J Am Stat Assoc 1984 79 387 649 652

[B42] Altman DG **Confidence intervals for the number needed to treat** BMJ 1998 317 7168 1309 1312 9804726 10.1136/bmj.317.7168.1309 PMC1114210

[B43] Andersen PK Klein JP Rosthöj S **Generalised linear models for correlated pseudo-observations, with applications to multistate models** Biometrika 2003 90 1 15 27

[B44] Andersen PK Hansen MG Klein JP **Regression analysis of restricted mean survival time based on pseudo-observations** Lifetime Data Anal 2004 10 4 335 350 15690989 10.1007/s10985-004-4771-0

[B45] Klein JP Logan B Harhoff M Andersen PK **Analyzing survival curves at a fixed point in time** Stat Med 2007 26 24 4505 4519 17348080 10.1002/sim.2864

[B46] Andersen PK Perme MP **Pseudo-observations in survival analysis** Stat Methods Med Res 2010 19 1 71 99 19654170 10.1177/0962280209105020

[B47] Jr FEH **Rms: Regression modeling strategies** 2013 R package version 3.6-3. [http://CRAN.R-project.org/package=rms]

[B48] R Core Team R: A Language and Environment for Statistical Computing 2012 Vienna: R Foundation for Statistical Computing [http://www.R-project.org/]

[B49] Gehrmann U Kuss O Wellmann J Bender R **Logistic regression was preferred to estimate risk differences and numbers needed to be exposed adjusted for covariates** J Clin Epidemiol 2010 63 11 1223 1231 20430578 10.1016/j.jclinepi.2010.01.011

[B50] Cheng S Wei L Ying Z **Analysis of transformation models with censored data** Biometrika 1995 82 4 835 845

[B51] Royston P Parmar MK **Flexible parametric proportional-hazards and proportional-odds models for censored survival data, with application to prognostic modelling and estimation of treatment effects** Stat Med 2002 21 15 2175 2197 12210632 10.1002/sim.1203

